# Exploring the anti-inflammatory potential of vitamin D in cardiometabolic diseases

**DOI:** 10.1016/j.metop.2025.100348

**Published:** 2025-01-09

**Authors:** Kabelo Mokgalaboni

**Affiliations:** Department of Life and Consumer Sciences, College of Agriculture and Environmental Sciences, University of South Africa, Calabash Building, Office no: 02-047 Florida Campus, 1710, South Africa

**Keywords:** Vitamin D, Inflammation, Monocyte chemoattractant protein-1, Tumour necrosis factor-alpha, Nuclear factor kappa beta, Cardiometabolic disease

## Abstract

The prevalence of cardiometabolic diseases is rising, and this is fuelled by inflammation, which tends to be worse in individuals with vitamin D (VD) deficiency. While non-steroidal anti-inflammatory interventions are available, they present with coagulation events. Hence, alternative therapy in the form of VD supplements is gaining research interest. This study reviewed the effect of VD supplementation on inflammation, focusing on nuclear factor kappa-beta (NF-κβ), tumour necrosis factor-alpha (TNF-α) and monocyte chemoattractant protein-1 (MCP-1) across different cardiometabolic disease. Thirty-seven studies, 16 rodent models and 21 clinical studies were evaluated. The study considered evidence from rodent models to understand the effect of VD on these markers of inflammation and its translatability to clinical studies. While the potential benefits of VD were notable in rodents, these effects were less consistent in clinical studies. Notably, rodent models showed a more pronounced impact of VD in reducing NF-κβ and TNF-α; however, clinical trials reported conflicting findings. Furthermore, the VD was important in reducing MCP-1 across different rodent models; this was partially demonstrated in clinical trials. Based on these findings, VD modulates inflammation in cardiometabolic disease by inhibiting the activation of NF-κβ and suppressing the production of TNF-α and MCP-1. Although VD has some possible benefits in rodent models, the translatability of these findings in clinical trials is limited. Hence, the presented evidence in this study calls for further randomised controlled trials to assess the effect of VD on inflammation in patients living with different conditions as a therapy to curb the inflammation and the risk thereof. Future trials should also focus on exploring the VD dose-response, optimal dose, and duration of VD intervention among these patients that may offer optimal benefits on inflammation.

## Graphical abstract

**Figure:**
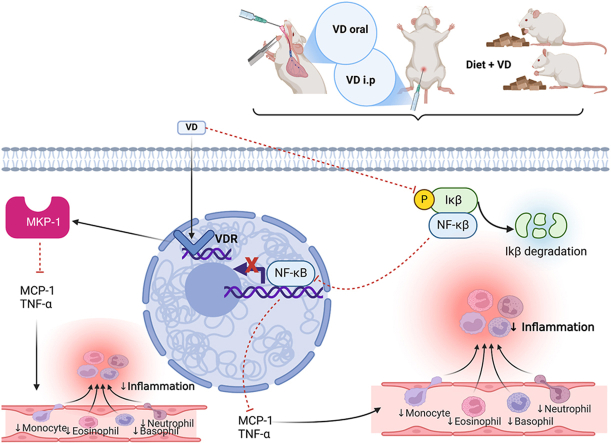

## 1 Introduction

Vitamin D deficiency (VDD), also known as Hypovitaminosis D, is a condition that occurs when there is an insufficient level of VD in the body [1]. This can result in various health complications, including bone health [2]. It is thus recognised globally as a health burden [3]. For instance, its global prevalence rate is 15.7 %, 47.9 %, and 76.6 % among participants with serum 25-hydroxyvitamin D levels less than (30, 50, and 75 nmol/l), respectively [4]. In Africa, 17.31 %, 34.18 % and 58.54 % have VD less than (30, 50, and 75 nmol/l), respectively [5]. In Asia alone, the prevalence of VDD is reportedly standing at 68 % [6]. Altogether, these statistics show that VDD is a global health concern.

**Fig. 1:**
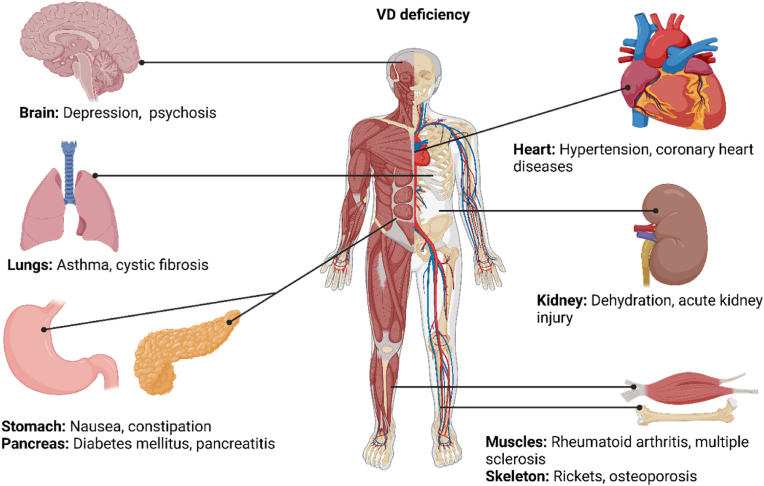
An overview of clinical manifestation in individuals with vitamin D deficiency (VDD). VDD causes complications that affect human systems, including nervous, cardiovascular, respiratory, urinary, digestive, muscular and skeletal. These encompass depression in the brain, hypertension, coronary heart diseases in the heart, asthma, and cystic fibrosis in the lungs, dehydration and acute kidney injury in the kidney, nausea and constipation in the stomach, diabetes mellitus and pancreatitis in the pancreas, arthritic and multiple sclerosis in the muscles and joints, rickets and osteoporosis in the skeletal system.

Therefore, there is a need to curb VDD, primarily as it is associated with several health complications. VD-related complications affect different body systems, including nervous, respiratory, digestive, cardiovascular, urinary, skeletal, and muscular systems (Fig. 1). Recently, it has been reported that VDD is associated with an increased triglyceride-glucose index (TyG) [7]. TyG is used as a surrogate marker for insulin resistance and to assess the risk of metabolic disorders like type 2 diabetes (T2D), cardiovascular diseases (CVD), and metabolic syndrome [[8], [9], [10]]. Therefore, its elevation in people with VDD suggests a substantial risk of CVDs and metabolic syndromes.

On the other hand, VDD promotes inflammation by disrupting the immune response, increasing the production of pro-inflammatory cytokines, impairing the function of Treg cells, and enhancing the activity of macrophages and dendritic cells [[11], [12], [13], [14]]. This imbalance can also contribute to the development and progression of metabolic disorders and CVD [15]. Inflammation exacerbates these conditions; therefore, controlling it would reduce the complications associated with VDD. Patients living with T2D have low VD and higher TNF-α; this suggests that VD supplementation could modulate inflammation by lowering the levels of TNF-α [16].

VD supplementation has been studied extensively and shown to have potential benefits in inflammation, especially in T2D. For example, MacGirlley has reported a significant decrease in proinflammatory markers, including tumour necrosis factor-alpha (TNF-α) and C-reactive protein (CRP) in T2D [17]. Notably, while this has been confirmed, there are still uncertainties about its effect on important transcription genes, such as nuclear factor kappa beta (NF-κβ), which regulates the production of various proinflammatory cytokines, including monocyte chemoattractant protein (MCP-1). Therefore, its control can be a target to control the production of proinflammatory and further regulate inflammation. For example, previous studies showed no effect of VD on NF-κβ [18,19]. MCP-1 is considered a significant maker of inflammation due to its contribution to the recruitment of monocytes and macrophages to the sites of inflammation, thus mediating the inflammatory response [20]. However, the effect of VD on this marker is inconsistent across various conditions. In studies among obese individuals, VD supplementation showed no effect on MCP-1 [21]. Due to conflicting results on MCP-1, TNF-α and NF-κβ, this review highlights the potential benefits of VD supplementation on markers and genes related to inflammation.

### 1.1 Absorption, bioavailability, and metabolism of vitamin D

**Fig. 2:**
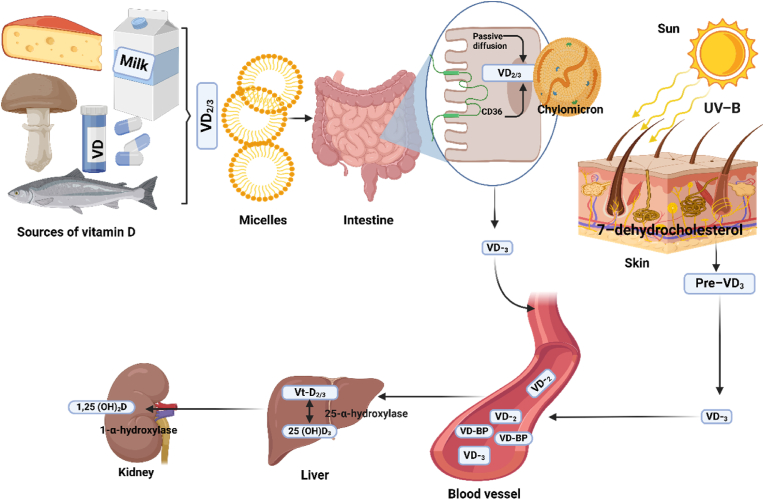
Synthesis, absorption, and metabolism of vitamin D. VD: vitamin D, VD-BP: vitamin D binding protein, CD36: cluster of differentiation 36. Absorption of VD is initiated by an intake of VD from the diet, a supplement directly from the interaction of the sun with the skin. The VD from diet and supplements is present in an inactive form (VD_2_ or _3_). These are taken by micelle into the small intestine through passive diffusion and scavenger receptors (CD36) into the enterocytes. Through chylomicrons, they are transported into the vascular system and bound to vitamin D binding protein (VD-BP). Similarly, pre-VD conversion from the skin through the sun produces VD3, which also enters the blood vessel by binding to the VD-BP.

An overview mechanism by which VD gets absorbed is summarised in Fig. 2. As a fat-soluble vitamin, its absorption requires dietary fats, digestion and emulsification of these fats to enhance its absorption [22]. Its absorption occurs in the small intestine, mainly the jejunum and ileum [23] (Fig. 2). When the dietary vitamins (D_2_ or D_3_) are consumed, as they reach the small intestine, the bile salts from the liver and gallbladder emulsify them, incorporating them into micelles. Micelles solubilise VD to enhance its absorption [24]. The VD is then taken up by the enterocytes in the small intestine either through passive diffusion or facilitated transport. Through passive diffusion, VD absorption occurs across the lipid membrane of enterocytes due to its lipophilic properties [23]. On the other hand, scavenger receptor B type I, CD36) promotes absorption when the body's VD level is reduced [25]. VD is incorporated into chylomicrons, triglycerides, and cholesterol inside the enterocytes to transport VD from the small intestine into the lymphatic system [26]. The chylomicrons enter the bloodstream via the lymphatic system, transferring VD from chylomicrons to VD-binding protein, a carrier protein in the blood that transports VD to target tissues, including muscle, liver, and adipose tissue (Fig. 2).

## 2 Brief methodology

### 2.1 Literature search

The search was done on PubMed, Web of Sciences, ResearchGate, and Semantic Scholar to identify relevant original studies from inception to November 28, 2024. The Boolean operators AND and OR were used to build the search. The search terms used included vitamin D, vitamin D_2_, and vitamin D_3_, MCP-1, monocyte chemoattractant protein-1, NF-κβ, nuclear factor kappa-beta, tumour necrosis factor-alpha, TNF-α. No language or duration restriction was applied in the search in all databases. Additional relevant studies were identified by manually screening the references for the literature.

### 2.2 Selection of studies

Only peer-reviewed studies were included, and grey literature (thesis, dissertations), conference abstracts, letters, and review papers were not considered. Randomised controlled, cross-sectional, observational, and clinical trials were included. Only studies that used VD in any treatment on selected inflammation markers were considered in this review. Studies were included if they reported the effect of VD on MCP-1, TNF-α, or NF-κβ in cardiometabolic diseases. Only thirty-seven studies [18,21,[27], [28], [29], [30], [31], [32], [33], [34], [35], [36], [37], [38], [39], [40], [41], [42], [43], [44], [45], [46], [47], [48], [49], [50], [51], [52], [53], [54], [55], [56], [57], [58], [59], [60], [61]] were relevant for this review: one included rodent models of prediabetes and prediabetes humans [62]. All studies were reviewed, and personal viewpoints and opinions were made based on the data presented in the original studies. A summary of findings from each study is presented in tabular format for preclinical and clinical studies. Data about the inflammatory markers was extracted from each clinical study, and fold increase or decrease was determined by dividing the baseline from post-treatment data.

## 3 Results

### 3.1 A description of the experimental studies is included

Seventeen preclinical studies [[27], [28], [29], [30], [31], [32], [33], [34], [35], [36], [37], [38], [39], [40], [41],^62^] included in this review were published between 2015 and 2024 (Table 1). These studies were published in ten countries globally. Four were published in China [[32], [33], [34],^62^], three in Iran [28,38,41], two from Indonesia [30,31], a France [29,35], and Egypt [39,61], and one from each of the following countries: Italy [40], Iraq [37], Korea [36], and Ukraine [27]. Different rodent strains such as Wistar, Spraque Dawley rats, C57BL/6, KKCg-AY/J and KKay mice were included in these studies.

**Table 1:** Effect of vitamin D on MCP-1, TNF-α and NF-κβ in animal models of cardiometabolic diseases.

Author	Experimental model	Country	Vitamin D dose and duration	Summary of experimental findings
Karkeni et al., 2015 [29]	C57BL/6J mice fed a high-fat diet (HFD).	France	15,000 IU/kg of VD_3_ for 4 days or 3000 IU/kg b.w for 10 weeks.	TNF-α mRNA decreased in HFD plus VD compared to HFD.
Farhangi et al., 2017 [28]	HFD-fed rats.	Iran	500 IU/kg by oral gavage alongside the rat's diet for 5 weeks.	TNF-α and MCP-1 in HFD plus VD were significantly lower than in the HFD group.
Zeng et al., 2017 [34]	Streptozotocin (STZ) and high-fat and high-glucose diets (HFHGD) induced diabetic nephropathy in Spraque Dawley rats.	China	Oral gavage with 0.03 μg/kg VD_3_ for 15 weeks.	VD-treated T2DN had reduced MCP-1 expression compared to T2DN.
Benetti et al., 2018 [40]	HFHGD-induced obesity in C57BL/6J mice.	Italy	7 μg/kg VD intraperitoneal injection (i.p) thrice a week for 2 months.	NF-κβ activation was reduced in HFHS treated with VD. No significant difference in TNF-α.
Sundari et al., 2020 [31]	Obese Wistar rats.	Indonesia	Oral administration of 2400 and 800 IU VD_3_ for 8 weeks.	mRNA MCP-1 was lower in VD groups compared to placebo.
Tian et al., 2020 [32]	KKCg-A^Y^/J and C57BL/6 mice.	China	Intragastric gavage of VD at 1.5, 3 and 6 μg/kg for 9 weeks.	No significant change in TNF-α in VD-treated groups.
Marziou et al., 2020 [35]	HFHGD-induced obesity in C57BL/6J mice.	France	HFHG supplemented with VD_3_ (5000 IU.kg^−1^) for 15 weeks.	Decreased mRNA expression of MCP-1 in the adipose tissue. No effect on TNF-α.
Rahimi et al., 2020 [41]	Obese Wistar rats.	Iran	VD was administered by an i.p of 0.5 μg daily for eight weeks.	No significant effect on NF-κβ gene expression following VD treatment.
Hamouda et al., 2021 [61]	STZ-induced diabetes in Wistar rats.	Egypt	500 IU VD and pioglitazone for weeks.	VD plus pioglitazone significantly reduced hepatic, TNF-α, and NF-κβ,
Mazanova et al., 2022 [27]	STZ-induced diabetes in Wistar rats.	Ukraine	Oral VD_3_ (600 IU/kg b.w.) for 30 days.	Decreased NF-κβ activation.
Ebrahimzadeh et al., 2022 [38]	HFD-induced obesity in rats.	Iran	500 IU/kg VD for 5 weeks.	There was a significant reduction in MCP-1 and NF-κβ in the VD-treated group compared to HFD without VD.
Chang et al., 2022 [36]	HFD-induced obesity in C57BL/6J mice.	Korea	HFD plus 1000 IU VD or HFD plus 10,000 IU VD for 16 weeks.	TNF-α, MCP-1 and NF-κβ were reduced in the treated group compared to the HFD group.
Krisnamurti et al., 2023 [30]	STZ plus HFHGD-induced prediabetes in Wistar rats.	Indonesia	VD_3_ at 100 IU/kg b.w, and one group received VD_3_ at 1000 IU/kg b.w for 12 weeks.	VD reduced the phosphorylation of NF-κβ p65.
Kmosh et al., 2024 [37]	White rats	Iraq	The 6th,7th and 8th groups received VD_3_ at 5000,10,000 and 15,000 IU for 21 days.	The higher dose led to a significant decrease in TNF-α compared to the lower dosages.
Zhang et al., 2024 [62]	KKay mice were fed HFD, and the C57BL/6J mice were fed a regular diet.	China	Low VD_3_ (0.42 IU/g/b.w), medium (1.68 IU/g/b.w), high (4.20 IU/g/b.w) for 16 weeks.	Moderate and high doses of VD reduced TNF-α compared to low doses.
Demerdash et al., 2024 [39]	HFD-induced obesity in Wistar rats.	Egypt	Oral gavage of 10 μg/kg VD or 10 μg VD plus 1 gm of calcium carbonate for 4 weeks.	Reduced TNF-α in both groups compared to the HFD group.
Gu et al., 2024 [33]	STZ-induced gestational diabetes (GDM) in C57BL/6J mice.	China	Oral VD 1.0 IU/g b.w for 18 days.	VD significantly reduced TNF-α and MCP-1.

T2DN: type 2 diabetic nephropathy; MCP-1: monocyte chemoattractant protein-1; STZ: streptozotocin; TNF-α:tumor necrosis factor-alpha; HFD: high-fat diet; NF-κβ: nuclear factor kappa beta; GDM: gestational diabetes.

### 3.2 The effect of vitamin D on inflammation, NF-κβ in rodent models

NF-κβ is a transcription factor complex that regulates cellular processes, including inflammatory responses [63]. NF-κβ in cytoplasm exists in an inactive form bound to the Inhibitor of κβ proteins (Iκβ) [64,65]. In response to stress, the Iκβ kinase (IKK) complex phosphorylates Iκβ [63,65]. This subsequently results in the degradation of Iκβ, releasing NF-κβ, which translocates to the nucleus and binds to DNA to activate gene transcription [[63], [64], [65]]. Further controlling the expression of cytokines TNF-α, IL-1β, IL-6 and MCP-1. The expression of these genes promotes inflammation. Therefore, the inhibition of NF-κβ would be targeted to control the inflammation by down-regulating their expression. In this review, the evidence showed that VD can reduce the activation of NF-κβ. A 30-day VD_3_ dose of 600 IU/kg b.w in streptozotocin (STZ)-induced diabetes in rats significantly reduced NF-κβ activation [27]. Similarly, 100 and 1000 IU/kg BW of VD_3_ in an STZ plus high-fat and high-glucose diet-induced prediabetes significantly reduced the phosphorylation of NF-κβ p65 [30]. This evidence is supported by other preclinical studies that revealed a significant decrease in NF-κβ post-VD treatment [35,36,38,40,61]. Therefore, reduced NF-κβ activation following VD supplementation suggests downregulation in the expression and production of inflammatory genes such as TNF-α, IL-6, and IL-1β, thus reducing inflammation. The same trend has also been noted in invitro, as murine macrophage and endothelial cells treated with VD significantly reduced the NF-κβ activity and mRNA and protein levels of Iκβα [19]. These results support the potential therapeutic effect of VD as an anti-inflammatory agent. However, recently, Rahimi has reported contradicting findings, where VD showed no impact on NF-κβ gene expression in obese rats [41]. It is important to highlight that this study used a very low dose of VD (0.5 μg).

### 3.3 The effect of vitamin D on inflammation, TNF-α in rodent models

TNF-α is a pro-inflammatory cytokine produced by macrophages and contributes to inflammation [66]. It promotes the production of other pro-inflammatory cytokines, such as IL-6, which recruit immune cells to the injury site [67]. The under-expression of TNF-α can also be a target to inhibit IL-6 production. In this study, the evidence from rodent models showed the potential benefits of VD in regulating TNF-α. Among the high-fat diet (HFD) fed rats, 500 IU/kg/day oral administration showed significantly lower TNF-α than HFD without treatment [28]. Additionally, in HFD-fed mice, treatment with VD led to a reduced mRNA of TNF-α decreased in HFD plus VD compared to HFD [29]. The same observation was seen in a mice model of gestational diabetes (GDM) when 1.0 IU/g of VD was administered [33]. Another study that used different dosages of VD showed that the effect may depend on the dosage administered, as only moderate and high doses of VD resulted in reduced TNF-α [62]. The findings are supported by other studies, which found similar findings [[34], [35], [36], [37],^61^]. On the other hand, other studies reported contrasting findings on TNF-α post-VD-treatment. For instance, Benetti et al. showed no effect of 7 μg/kg intraperitoneal injection of VD on TNF-α in C57BL/6J mice. A similar finding was observed in a study by Ref. [35]. Consistently, another study [32] showed no effect of VD on TNF-α in KKCg-A^Y^/J and C57BL/6. This study administered doses of VD (1.5, 3 and 6 μg/kg), all showing no effect on TNF-α. This could also be attributed to the resilience of TNF-α signalling, insufficient dose, intervention duration or the genetic characteristics of the KK-Ay mice model. The persistence of TNF-α despite VD treatment could reflect its involvement in the pathogenesis of T2D in this model. It is worth highlighting that the KK-Ay mice model used in the study is genetically susceptible to obesity and diabetes [68], characterised by chronic low-grade inflammation, which might not be reversible by anti-inflammatory interventions. Despite anti-inflammatory interventions, TNF-α remains high in inflammatory states due to constant immune activation [69].

### 3.4 The effect of vitamin D on inflammation, MCP-1 in rodent models

MCP-1 is a chemokine that is important in inflammatory and immune responses [70]. Activated monocytes and macrophages produce MCP-1 to recruit more immune cells to the injury site [71]. In T2D and obesity, MCP-1 production increases in adipose tissues, promoting chronic low-grade inflammation [71,72]. The elevated levels or expression of MCP-1 signals state of inflammation. The evidence from experimental studies revealed the potential benefits of VD on MCP-1, as demonstrated by a significant decrease in the mRNA expression and levels of MCP-1 across different rodent models of prediabetes, obesity and diabetes mellitus. In HFD-fed rats given an oral dose of 500IU/g of VD, there was a significant decrease in MCP-1 compared to the HFD group without treatment [28]. Similarly, in C57BL/6 HFHG-fed diet mice, the administration of 5000 IU/g VD led to a significant decrease in mRNA expression of MCP-1 [35]. Another study consistently showed that administration of VD, even at a low dose (1.0 IU/g) in GDM C57BL/6J mice, reduces the level of MCP-1 [33]. Among the Wistar obese rats, the administration of either 2400 IU/g or 800 IU downregulated the mRNA expression of MCP-1 [31]. Zeng et al., 2017 also demonstrated a significant reduction in MCP-1 following 0.03 IU/g VD supplementation in diabetic nephropathic rats [34]. This evidence is supported by findings from obese rats, where 500 IU of VD reduced MCP-1 [38]. Chang et al., 2022 also found similar findings in C57BL/6J mice [36]. Altogether, this evidence supports using VD as an anti-inflammatory agent as it substantially reduces the expression of proinflammatory genes and levels of cytokines thereof.

### 3.5 The description of clinical studies included

Of the included studies, twenty-one [18,21,[42], [43], [44], [45], [46], [47], [48], [49], [50], [51], [52], [53], [54], [55], [56], [57], [58], [59],^62^] were conducted in humans with different cardiometabolic diseases, including T2DM, obesity, chronic kidney disease, diabetic nephropathy, and dyslipidemia. All studies were randomised controlled trials except for one [21] that used a quasi-experimental design. The mean age range was 23–66.9 years (Table 2). These studies were conducted in ten countries, including Australia [18,48], China [45,62], Denmark [46,51], Finland [58], Germany [21], Iran [44,50,[52], [53], [54],^56^,^57^], Lebanon [49], Turkey [59], Saudi Arabia [55] and the United States of America [42,43,47]. The duration of vitamin intervention ranged from 1 to 40 weeks, with the highest dose being 250,000 IU (Table 2).

**Table 2:** Effect of vitamin D on MCP-1, TNF-α and NF-κβ in cardiometabolic disease.

Author	Design	Country	Sample	Age	Vitamin D	Findings on inflammation
Alvarez et al., 2013 [43]	Double-blind, randomised, placebo-controlled trial	USA	46 patients with CKD, 22 on VD_3_, and 24 on placebo.	62.5 ± 9.6	50,000 IU VD_3_ weekly for 12 weeks, followed by 50,000 IU VD_3_ weekly for 40 weeks.	One year of VD treatment significantly decreased MCP-1.
Wamberg et al., 2013 [46]	Double-blind, placebo-controlled randomised clinical trial	Denmark	Obese adults, 22 on cholecalciferol and 21 on placebo.	39.5 ± 8.0 41.2 ± 6.8	A daily dose of 7000 IU of VD_3_ for 26 weeks.	Significant increase in MCP-1 at the end of VD compared to baseline.
Gagnon et al., 2014 [48]	Double-blinded, placebo-controlled trial	Australia	Prediabetes patients, 35 on VD and 45 on placebo.	53.8 ± 11.9 55.3 ± 11.1	2000–6000 IU of VD_3_ for six months.	There was no significant difference in TNF-α in the VD and placebo.
Ghavamzadeh et al., 2014 [54]	Double-blinded randomised, placebo-controlled trial	Iran	T2D patients, 26 on VD and 25 on placebo.	52.3 ± 2.1 49.3 ± 2.0	400 IU VD_3_ for 14 weeks	Significant decrease in TNF-α post-treatment compared to baseline.
Kampmann et al., 2014 [51]	Double-blind, randomised, placebo-controlled trial	Denmark	T2D patients, seven on VD and eight on placebo.	61.6 ± 4.4 57.0 ± 4.5	VD_3_ (280 μg daily for 2 weeks, 140 μg daily for 10 weeks).	There is no significant difference in TNF-α in VD compared to placebo.
Tuomainen et al., 2015 [58]	Double-blinded randomised placebo-controlled trial	Finland	Prediabetes, 45 on VD_3_ and 21 on placebo.	65.7 ± 7.0	40 or 80 μg of VD_3_ for 5 months.	There were no significant changes in TNF-α in all treated groups compared to placebo.
Duggan et al., 2015 [47]	Randomised clinical trial	USA	218 postmenopausal, overweight or obese 94 on VD, 93 on placebo.	60.3 ± 5.3 59.0 ± 4.7	2000 IU/day oral vitamin D_3_ for 12 months.	No significant difference in TNF-α in the VD group.
Al-sofiani et al., 2015 [55]	Double-blinded randomised placebo-controlled trial	Saudi Arabia	T2D patients, 10 on VD and 10 on placebo.	54.8 ± 9.2 55.0 ± 11.9	5000 IU VD_3_ for 12 weeks.	No significant changes in TNF-α post-VD compared to baseline.
Mousa et al., 2017 [18]	Parallel-group, double-blind, randomised, placebo-controlled trial	Australia	55 overweight/obese, 26 on VD and 28 on placebo.	30.5 ± 2.8 29.5 ± 4.0	An initial bolus dose of 100,000 IU (in 2 capsules) followed by 4000 IU (in 4 capsules) of VD_3_ daily for 16 weeks.	There is no difference in TNF-α, MCP-1 and NF-κβ in VD and placebo.
Sosale et al., 2018 [57]	Randomised-parallel group-opened labelled controlled trial	India	T2D and dyslipidemia patients, 29 on VD and 31 on placebo	53 (48–60) 55 (50–61)	60,000 IU once a week for 8 weeks, followed by once a month for 4 months.	No significant changes in TNF-α post-VD treatment.
Esfandiari et al., 2019 [56]	Double-blinded randomised placebo-controlled trial	Iran	Diabetic nephropathy, 25 on VD and 25 on placebo.	39.7 ± 7.3 47.3 ± 6.1	VD_3_ at 50,000 IU for 8 weeks.	Significant decrease in TNF-α post-VD treatment compared to baseline.
Omidian et al., 2019 [44]	Parallel randomised double-blind placebo-controlled clinical trial	Iran	23 T2DM on VD and 23 on placebo.	51.3 ± 4.7 52.4 ± 5.7	One VD tablet (4000 IU) for three months (13 weeks).	MCP-1 decreased in the VD group post-treatment and when compared to placebo.
Hajj et al., 2020 [49]	Randomised controlled double-blind	Lebanon	T2D patients, 45 on VD and 43 on placebo.	66.9 ± 4.1 65.7 ± 4.5	30,000 IU VD_3_ weekly for six months.	Significant decrease in TNF-α post-VD treatment.
Imanparast et al., 2020 [52]	Randomised-placebo-controlled trial	Iran	T2D patients, 23 on VD3, 23 on placebo.	53.6 ± 12.3 51.7 ± 9.1	50,000 IU VD_3_ for 4 months.	A significant decrease in TNF-α post-VD treatment compared to baseline.
Kallantar et al., 2020 [53]	Randomised controlled trial	Iran	103 overweight women.	29.2 ± 3.2 27.4 ± 1.4	1000 IU VD for 8 weeks	There is no significant difference in TNF-α post-treatment compared to baseline.
Wiciński et al., 2021 [21]	Quasi-experimental	Germany	33 obese patients, 16 women and 17 men.	23–71	VD was administrated at a dose of 2000 IU for 3-months.	There is no difference in MCP-1 before and after VD supplementation.
Khodadoust et al., 2021 [50]	Quasi-experimental	Iran	Overweight men,13 in pilates exercise plus VD and 13 in the control group.	49.5 ± 2.4 49.0 ± 3.3	50,000 IU VD for 8 weeks	A group on pilates exercise plus VD significantly decreased TNF-α post-treatment compared to baseline.
Ağar et al., 2022 [59]	Prospective interventional study	Turkey	25 overweight/obese women with PCOS	NR	2000 IU VD for 12 months.	Significant decrease in NF-κβ post-VD treatment.
Gu et al., 2022 [45]	Randomised controlled trial	China	T2D patients, 86 on VD and 92 on placebo.	NR	400 IU VD daily for 90 days.	VD significantly decreased MCP-1.
Zhang et al., 2024 [62]	Randomised, placebo-controlled trial	China	Prediabetes patients, 30 on VD_3_ and 30 on placebo.	58.4 ± 7.2 58.2 ± 7.1	VD_3_ at 1600 IU/day for 24 weeks.	There was no significant difference in TNF-α in VD and placebo.
Nadeem et al., 2024 [60]	Cross-sectional	Pakistan	50 T2DN on VD and 50 T2DN without treatment.	51.3 ± 0.9 50.0 ± 1.5	Oral adjunct VD therapy.	VD decreased TNF-α in diabetic nephropathy compared to the group without treatment.

T2DN: type 2 diabetic nephropathy; T2D: type 2 diabetes; CKD: chronic kidney disease; PCOS: polycystic ovarian syndrome, MCP-1: monocyte chemoattractant protein-1; TNF-α: tumour necrosis factor-alpha; NF-κβ: nuclear factor-kappa beta, NR: not reported.

### 3.6 The effect of vitamin D on inflammation, NF-κβ in clinical studies

Only two studies reported the effect of VD on NF-κβ in obesity. One among obese/overweight women with PCOS showed a significant reduction in the expression of NF-κβ, which was a 2.95 decrease post-VD treatment (p = 0.01) [59] (Table 3). Yet another trial showed an observable decrease (1.17 folds) in the activity of NF-κβ when the baseline was compared to post-VD treatment (p = 0.05) [18]. These two studies included overweight/obese participants, known to have high inflammation induced by adipocytes [73]. Additionally, the differences could have arisen from the employed different intervention durations as Ağar et al., 2022 [59] extended intervention to at least 12 months while Mousa [18] completed the study in 16 weeks. It is also worth noting that a later trial used a VD-deficient obese population; thus, the supplemented dosages might not have been sufficient to offer a potential effect on inflammation. Additionally, in this parallel trial, the dosage was reduced from 100,000–4000 IU, which might have affected the efficacy of the treatment. Furthermore, the results may be attributed to the small sample size, which failed to achieve statistical power to identify changes in this inflammatory marker. Despite the observational difference noted in this study between the baseline and post-treatment (1.17-fold decrease), the effect was not significant, hence calling for more research on this population. There is a limitation in evidence investigating the impact of VD on NF-κβ, as noted by only two trials.

**Table 3:** Comparative effect of vitamin D on different markers of inflammation in patients with cardiometabolic diseases.

Author	NF-κβ			TNF-α			MCP-1		
	Baseline	Post	P	Baseline	Post	P	Baseline	Post	P
Alvarez et al., 2013 [43]	–	–	–	−2.8 (−10.6, 2.1)	1.1 (−5.6, 1.6)	>0.05	−6.2 ± 13.3	−3±14.5	<0.10
Wamberg et al., 2013 [46]	–	–	–	–	–	–	112.9 (91.5; 135.1)	127.3 (106.7, 146.1)	0.04
Gagnon et al., 2014 [48]	–	–	–	6.13 ± 2.18	6.60 ± 1.43	>0.05	–	–	–
Ghavamzadeh et al., 2014 [54]	–	–	–	10.43 ± 1.12	4.89 ± 1.24	0.001	–	–	–
Kampmann et al., 2014 [51]	–	–	–	NS	NS	>0.05	–	–	–
Tuomainen et al., 2015 [58]	–	–	–	2240 ± 480	−15.8 ± 265.1 at 40 μg 42.5 ± 312.7 at 80 μg	0.635	–	–	–
Duggan et al., 2015 [47]	–	–	–	9.3(8.45, 10.24)	9.14 (8.31, 10.05)	0.41	–	–	–
Al-sofiani et al., 2015 [55]	–	–	–	53.87 (51.46, 62.27)	51.46 (47.83, 57.48)	0.74	–	–	–
Mousa et al., 2017 [18]	33.6 (26.6–63.6)	28.6 (23.2–35.1)	0.05	29.6(14.6,55)	24.3(11.1, 45.6)	0.3	625(398.4, 951.4)	554.1(429.6, 925.6)	0.6
Sosale et al., 2018 [57]	–	–	–	9.16 (7.41, 9.63)	−3.68 (−4.37, 2.99)	>0.05	–	–	–
Esfandiari et al., 2019 [56]	–	–	–	136.6 ± 33.65	118.47 ± 27.58	0.002	–	–	–
Omidian et al., 2019 [44]	–	–	–	–	–	–	240.2 ± 27.6	197.6 ± 12.7	<0.02
Hajj et al., 2020 [49]	–	–	–	3.05 ± 1.02	2.61 ± 1.04	0.0001	–	–	–
Imanparast et al., 2020 [52]	–	–	–	4.16 ± 3.68	2.43 ± 1.54	0.012	–	–	–
Kallantar et al., 2020 [53]	–	–	–	0.95 ± 0.1	0.91 ± 0.08	>0.05	–	–	–
Wiciński et al., 2021 [21]	–	–	–	–	–	–	230.35 ± 47.83	246.41 ± 47.8	0.157
Khodadoust et al., 2021 [50]	–	–	–	7.47 ± 1.26	5.73 ± 1.11	<0.001	–	–	–
Ağar et al., 2022 [59]	3.22 ± 1.09	1.10 ± 0.30	0.01	–	–	–	–	–	–
Gu et al., 2022 [45]	–	–	–	–	–	–	51.11 ± 20.86	25.42 ± 13.06	<0.0001
Zhang et al., 2024 [62]	–	–	–	2.23 ± 0.4	2.27 ± 0.07	0.611	–	–	–
Nadeem et al., 2024 [60]	–	–	–		VD:1.47 ± 0.044^a^ Placebo:28.95 ± 0.75^a^	<0.05	–	–	–

a: VD vs no treatment, –: no data was reported, NS data not sufficient as reported graphically, MCP-1: monocyte chemoattractant protein-1, TNF-α: tumour necrosis factor-alpha, NF-κβ: nuclear factor kappa-beta.

Data presented as mean ± SD or median and range.

### 3.7 The effect of vitamin D on inflammation, TNF-α clinical studies

Fourteen studies included in this review assessed the effect of VD on TNF-α in cardiometabolic diseases. However, discordant results were reported across these studies. Only six studies showed a significant decrease in TNF-α post-VD treatment compared to baseline or placebo groups [49,50,52,54,56,60]. Specifically, the administration of VD at 30,000 IU led to a decreased (1.17-fold) TNF-α in T2D [49]. A more pronounced benefit was reported by Gavhamzadeh, where 400 IU of VD_3_ in T2D led to a significant 2.13-fold decrease in TNF-α post-treatment [54]. However, other trials demonstrated contradicting results that showed no effect of VD on TNF-α [18,47,48,51,55,57,58,62]. While Sosale et al. [57] reported no significant effect of VD on TNF-α, it is worth noting that there was a 2.5-fold decrease in this marker post-treatment. The evidence from these trials suggests an anti-inflammatory limitation of VD on TNF-α. Another reason for this limitation could be the baseline TNF-α level; the higher the baseline TNF-α, the lesser the effect of VD post-treatment. For instance, Mousa [18] reportedly recruited patients with VD less than 50 ng/mL, and this negatively affected the overall potential efficacy of supplemented VD.

### 3.8 The effect of vitamin D on inflammation, MCP-1 clinical studies

Six of the 21 included studies reported the effect of VD on MCP-1 in cardiometabolic diseases. However, there were discordant reports from these studies regarding VD impact; some showed null findings, and others reported an elevation. In contrast, other studies showed a reducing potential of VD on MCP-1. Despite their varying doses, those that showed a decrease in MCP-1 were conducted in T2D and CKD patients [[43], [44], [45]]. In a study by Guo et al. [44], there was a notable 2.01-fold decrease in MCP-1 post-VD treatment among T2D patients (Table 3). In contrast, another trial demonstrated no effect of VD on MCP-1 [18]. Similarly, according to Wiciński of 2021 [21], three months of administration of 2000 IU VD in T2D had no effect on MCP-1. However, different results were reported in a trial that used obese participants, where 7000 IU VD supplementation resulted in persistent inflammation, as shown by an increase (0.88-fold) in MCP-1 post-treatment [46]. In this trial, VD_3_ was administered to obese patients with VD levels below 50 nmol/l regarded as VDD. Therefore, VD supplementation in obese patients with VDD must be taken with caution to prevent the risk of inflammation and to understand its potential effect better. The researcher also specified that their results may be at risk of statistical type 2 errors due to limited sample size. Furthermore, as obesity and its associated complications develop over the years, treatment for 26 weeks might be too short for any notable potential reversal effects in metabolic aberrations.

## 4 Discussion

The prevalence of cardiometabolic disease associated with chronic inflammation is consistently rising due to increasing rates of obesity, ageing, and urbanisation [74]. The prevalence of VDD is also highly alarming, and this is worse when in co-existence with subclinical inflammation [75]. Acute inflammation interventions such as aspirin reduce inflammation by inhibiting cyclooxygenase-2 (COX-2). However, their use is associated with bleeding, which can be fatal in some cases [76,77]. This calls for urgent research to find alternative therapies to prevent and manage these cardiometabolic conditions. Interestingly, the use of supplements has been promoted in different clinical settings. Supplements provide precise doses of anti-inflammatory nutrients that may be difficult to obtain in sufficient quantities through diet alone. They are well-tolerated with less adversity when properly used. In this study, the beneficial effect of VD supplementation was observed in the experimental models of cardiometabolic disease, modulating different markers of inflammation, mainly the NF-κβ, TNF-α, and MCP-1. However, the translatability of the findings from rodent studies was limited, as the results were not fully reproduced in clinical studies. Although other clinical trials showed positive results, it is worth noting that there are potential limitations that could have led to discordant results in these conditions (CKD, obesity, T2D, and prediabetes). All these conditions have different pathophysiologies, which might exacerbate inflammatory status even post-VD treatment. For instance, obesity is known to have inflammation induced by adipocytes, which might persist even after anti-inflammatory interventions [73].

It is also important to point out that different forms of VD, doses, and duration of intervention were applied in these studies, which could also play a significant role in the effectiveness of VD treatment. The moderate dosages (2000 IU) and the VD showed no impact on MCP-1 among obese patients in a quasi-experimental design [21]. Mousa et al., 2017 [18] also investigated the effect of VD on inflammation in obese patients; in this parallel trial, the VD was initially administered as a bolus dose of 100,000 IU followed by 4000 IU. However, there was no effect on TNF-α, MCP-1 and NF-κβ. Of concern is that even at lower doses, VD revealed no impact on TNF-α [51,53,55,57,58,62]. Another contradicting finding reported by Wamberg et al., 2013 [21] was an increased MCP-1 following 26 weeks of VD_3_ treatment in obese individuals. These contradicting results may be because the obese patients in this study had low VD levels (< 50 nmol/L); hence, the administered dosage (7000 IU) was not adequate to offer anti-inflammatory properties. In this trial, VD was administered daily to obese patients for 26 weeks, which might be too short for the observation of optimal effect, especially in states of obesity.

Despite all the limitations reported above, other studies have shown the potential benefits of VD on markers of inflammation, including TNF-α and MCP-1. Interestingly, VD reduced TNF-α in T2D patients [49]. Consistently, MCP-1 was reportedly reduced post-VD treatment in other studies [[43], [44], [45]]. These results partly support what has been reported in preclinical studies suggesting the potential effect of VD as an anti-inflammatory agent across various conditions. MCP-1 is a marker of inflammation due to its role in recruiting immune cells to injury sites [20,78]. It also binds to the C-C chemokine receptor type 2 (CCR2) on monocytes, thus promoting their migration into the inflamed tissues to initiate inflammatory responses [20]. Therefore, reducing the level of MCP-1 in the circulation can be considered an essential step in ameliorating inflammation in different metabolic conditions.

**Fig. 3:**
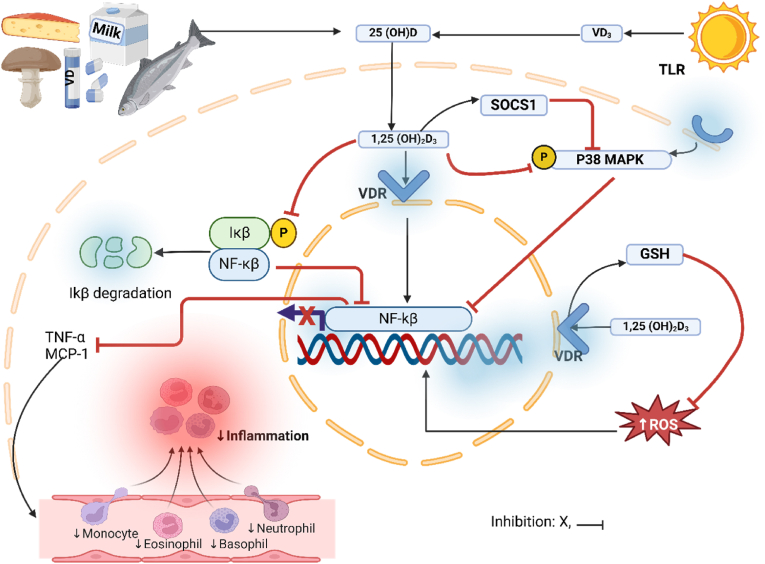
Possible pathway by which vitamin D improves inflammation. An active form of VD (1.25 (OH)_2_D_3_ activates the suppressor of cytokine signaling-1 (SOCS1), and an active SOCS1 inhibits the activation of mitogen-activated protein kinase p38 (MAPK), which promotes transcription of NF-κβ. VD can also inhibit the p38 MAPK. Therefore, the inhibition of p38 MAPK blocks the transcription of NF-κβ in the nucleus. On the other hand, VD inhibits the phosphorylation of Iκβ, further inhibiting the transcription of NF-κβ. Altogether, inhibition of NF-κβ leads to suppressed TNF-α alongside MCP-1 production, reducing monocyte recruitment. Altogether, these reduce inflammation. VD: vitamin D, VDR: vitamin D receptor, TLR: toll-like receptor, NF-κβ: nuclear factor kappa beta, TNF-α: tumour necrosis factor-alpha, MCP-1: monocyte chemoattractant protein-1, Iκβ: inhibitor of kappa beta, SOCS1: suppressor of cytokine signaling 1, MAPK: mitogen-activated protein kinase, GSH: glutathione, ROS: reactive oxygen species. Adapted from Ding et al., 2013 [82].

The mechanism by which VD lowers MCP-1 is complex and involves various pathways. For instance, VD inhibits lipopolysaccharide (LPS)-induced MCP-1 expression in human monocytes through downregulation of the p38 mitogen-activated protein kinase (MAPK) signalling pathway [79,80]. On the other hand, VD promotes the expression of MAPK phosphatase-1 (MKP-1). An upregulation of the latter enzyme dephosphorylates and further reduces the activation of p38 MAPK, resulting in decreased production of MCP-1 [80,81]. Furthermore, VD inhibits the NF-κβ pathway, thus reducing MCP-1 levels [82]. Moreover, VD supplementation has been associated with increased levels of antioxidants, including glutathione (GSH) [45,60,83]. VD upregulates the expression of genes that promote GSH synthesis, such as glutamate reductase and glutamate-cysteine ligase, increasing GSH production [84,85]. Conversely, an increased level of GSH suppresses the production of MCP-1 by reducing ROS that promotes MCP-1 expression through MAPK kinases [86] (Fig. 3). Altogether, these interactions between VD, GSH, and inflammation suggest that supplementation with VD can ameliorate MCP-1-mediated inflammation. VD promotes the expression of Iκβα, an inhibitor of NF-κβ, stabilising its mRNA and reducing its phosphorylation, resulting in reduced translocation of NF-κβ into the nuclear for transcription [19]. Conversely, VD reduces TNF-α by inhibiting the expression of other genes associated with its production. Firstly, its active form, 1,25 dihydroxyvitamin D, inhibits the NF-κβ pathway. This is achieved by inhibiting phosphorylation and degradation of Ikβ proteins, which primarily release NF-κβ to initiate transcription [87] (Fig. 3). As a result of the presence of VD, the resulting free-floating NF-κβ in the cytoplasm reduces the expression of proinflammatory cytokines, including TNF-α. VD also binds to VD receptors, resulting in a VD-VDR complex that controls gene expression and inhibits NF-κβ activation [87]. This complex downregulates genes that promote inflammation and upregulates those that counteract inflammation [88]. These subsequently reduce the production of TNF-α and ameliorate inflammation. Other researchers have also shown that VD can ameliorate inflammation through modulation of immune cells [89]. VD stimulates Tregs differentiation and function, which inhibits proinflammatory response [90]. Elevated Tregs result in low levels of TNF-α due to their role in the production of anti-inflammatory cytokines such as IL-10 [91]. VD also downregulates the expression of TLRs on immune cells, which participates in the recognition of pathogens and initiation of inflammatory response [92]. This activity inhibits the activation of NF-κβ and the production of TNF-α. Other scholars have also reported that VD inhibits COX-2-mediated inflammatory response through modulation of the Akt/NF-κβ signalling pathway by directly upregulating thioesterase superfamily member-4 [93]. While there is a limitation in the translatability of preclinical findings clinically in cardiometabolic disease, the overall evidence gathered in this study suggests the potential of VD as an anti-inflammatory agent in these cardiometabolic diseases. We also noted a skewness in the publication of these studies; most of the studies were published in Asia (62.2 %), 21.6 % in Europe, and 5.4 % in Africa, America, and Oceania. However, the concern was less representative of Africa, America, and Oceania, as only two studies were found in one country across these three continents. The presented findings in this study should be interpreted with caution as they are not representative of the global picture due to publication bias.

## 5 Conclusion and future directions

The evidence summarised in this review supports the beneficial effect of VD as a supplement in improving inflammation in rodent models of cardiometabolic disease. These studies showed that VD inhibited the activity of prominent markers of inflammation, such as NF-κβ, TNF-α, and MCP-1 in rodent models. Interestingly, the results were partially reproduced in clinical trials, further confirming the beneficial effect of VD against inflammation in different cardiometabolic conditions. Although the benefits were observed in clinical studies, potential limitations were noted due to the discordant findings. Additionally, lack of evidence, especially on NF-κβ, may have limited the interpretation of the summarised clinical evidence. Different modes of VD administration, doses, durations, and various cardiometabolic conditions may also have led to different findings reported in this study. The translatability of rodents to clinical findings may also be due to the different mechanisms and modes of VD absorption. All these limitations warrant future-powered large-scale randomised controlled trials with proper methodological quality to focus on the VD dose-response relationship to confirm and validate the translatability of evidence from preclinical studies in cardiometabolic disease clinically, especially on NF-kβ. Also, the optimal dose of VD and duration of intervention must be standardised to assess the effect on these markers.

### 5.1 Clinical implications

The anti-inflammatory properties of VD support its use as an adjunctive therapy to reduce inflammation in patients with cardiometabolic diseases, thus improving outcomes when combined with other proven therapies. VD supplementation could be explored in patients with elevated inflammatory markers or early-stage disease to determine its efficacy in modulating inflammation. The findings may be used as a baseline when developing personalised nutrition plans that include VD to reduce inflammation, especially in individuals at high risk of cardiometabolic complications. Despite the promising preclinical evidence, the translation of these effects into clinical outcomes remains uncertain due to variability in study design and patient characteristics in clinical trials. Addressing these limitations in future trials is critical.

## Data availability statement

Not applicable.

## Funding

This research was partially funded by the NRF Special Transformation Awards (NRF Awards) (NSTA231114163486), Research Development Grants for nGAP Scholars (NGAP23022780506), and the Black Academics Advancement Programme PhD Track (NFSG230512105121). The funders had no role in the design of the study, in the collection, analysis, or interpretation of data, in the writing of the manuscript, or in the decision to publish the results.

## Conflict of interest

Not applicable.
